# Aligning Leader Behaviors With Innovation Requirements Improves Performance: An Experimental Study

**DOI:** 10.3389/fpsyg.2020.01332

**Published:** 2020-07-07

**Authors:** Friederike Gerlach, Katharina Heinigk, Kathrin Rosing, Hannes Zacher

**Affiliations:** ^1^ Psychology of Entrepreneurial Behavior, Institute of Psychology, University of Kassel, Kassel, Germany; ^2^ Work and Organizational Psychology, Institute of Psychology – Wilhelm Wundt, Leipzig University, Leipzig, Germany

**Keywords:** innovation requirements, opening leader behavior, closing leader behavior, creativity, implementation

## Abstract

In this experiment, the effect of the alignment of leader behaviors with innovation requirements was investigated. A sample of *N* = 245 students participated in a laboratory experiment. Participants had to address either creativity or implementation requirements within a task and received a leadership manipulation in a video message. Results showed that the alignment of leader behaviors with innovation requirements led to improved performance. These findings contribute to the literature by addressing the specific requirements within the innovation process and by showing that aligning leader behaviors with these requirements contribute to performance in the innovation process.

## Introduction

Innovation of central importance to organizations as they strive to gain or maintain a competitive advantage in the market (Porter, 1990; Shalley and Gilson, 2004; Rosenbusch et al., 2011). *Innovation* is defined as the “intentional introduction and application within a role, group, or organization of ideas, processes, products, or procedures, new to the relevant unit of adoption” (West and Farr, 1990). This definition speaks to the complexity of the innovation process in that it incorporates both a requirement to be creative and a requirement to implement (West and Farr, 1990; Potočnik and Anderson, 2016). Creativity requirements are present when employees need to generate new and creative ideas (Amabile, 1988; West and Farr, 1990; Amabile and Pratt, 2016). Implementation is required when the realization of an outcome is needed (West and Farr, 1990; West, 2002a,b). Innovation scholars have emphasized the difficulty of integrating creativity and implementation and thus, both requirements have implicitly been recognized (Smith and Tushman, 2005; Bledow et al., 2009; Miron-Spektor and Erez, 2017). As creativity and implementation are very different, sometimes even contradictory, they need to be addressed by different behaviors within the innovation process (Bledow et al., 2009; Rosing et al., 2011; Miron-Spektor et al., 2018). We propose that aligning behavior with creativity and implementation requirements is crucial for both employees and leaders to successfully accomplish innovation outcomes. Nonetheless, most empirical research on innovation processes has neglected this differentiation within the process (West, 2002a; Peralta et al., 2015; Jankowska et al., 2018). Thus, conceptualizing creativity and implementation requirements as key parts of the innovation process will enable us to uncover the effectiveness of leader behaviors in specific situations within the innovation process (Shalley et al., 2004; Rosing et al., 2011) and thereby contribute to a better understanding of the relationship between leadership and innovation outcomes.

Leadership has been proposed as one of the central influences within the innovation process, and different models such as transformational leadership and leader-member exchange have been found to be relevant in this regard (Hammond et al., 2011; Junni et al., 2015; Hughes et al., 2018). However, most leadership models have overlooked creativity and implementation requirements within the innovation process. We suggest that research on leadership and innovation will benefit from an integration of a micro-level perspective on these requirements. For this purpose, a contingency perspective looking at different leader behaviors depending on the situational requirements is necessary (Fiedler, 1971; Peters et al., 1985; Rosing et al., 2011). The model of ambidextrous leadership provides such a perspective and addresses both situational requirements by proposing two types of leader behavior (Rosing et al., 2011). *Opening leader behavior* entails leaders encouraging their followers to take risks and giving opportunities for independent thinking and experimenting with diverse ideas and should be especially helpful in situations when creativity is required (Rosing et al., 2011). In contrast, *closing leader behavior* involves leader actions such as ensuring rules are followed, establishing routines, and monitoring target attainments and should increase follower performance when implementation is required (Rosing et al., 2011). The importance of aligning leader behavior to the requirements becomes more apparent when considering the possible effects of a misalignment between requirements and behaviors. For example, a leader who focuses on meeting deadlines (closing leader behavior) when the task requires the development of new ideas (creativity requirement) or encourages thinking in new directions (opening leader behavior) when the task requires the final realization of a product (implementation requirement) is unlikely to be successful. This example illustrates that showing opening and closing leader behaviors is not enough, but that these leader behaviors need to be aligned with situational requirements to promote innovation performance. This underlines the importance of a contingency perspective, where the relevant leader behavior depends on the tasks relevant to the situation (Fiedler, 1971; Rosing et al., 2011). Therefore, the alignment of leader behavior and situational requirements is at the heart of the ambidextrous leadership model (Rosing et al., 2011). Yet, interestingly, this crucial idea of the model has not been investigated empirically.

Moreover, most research on leadership in innovation relied on correlational data (Hughes et al., 2018). Unfortunately, we cannot draw causal conclusions concerning the influence of leadership on innovation based on these studies (Hülsheger et al., 2009; Antonakis, 2017; Hughes et al., 2018). Therefore, this research contributes by analyzing the causal effects of leader behaviors on innovation performance. This is important as a reverse effect could also exist (Antonakis et al., 2010, 2014; Hughes et al., 2018). Furthermore, there has been considerable debate on the influence of perception on self-report measures of leadership and innovation performance (Reiter-Palmon et al., 2012; Antonakis, 2017; Hughes et al., 2018). For example, research shows that knowledge about leaders’ performance influences the evaluation of leader behaviors (Lord et al., 1978; Wang et al., 2019).

**Figure 1:**
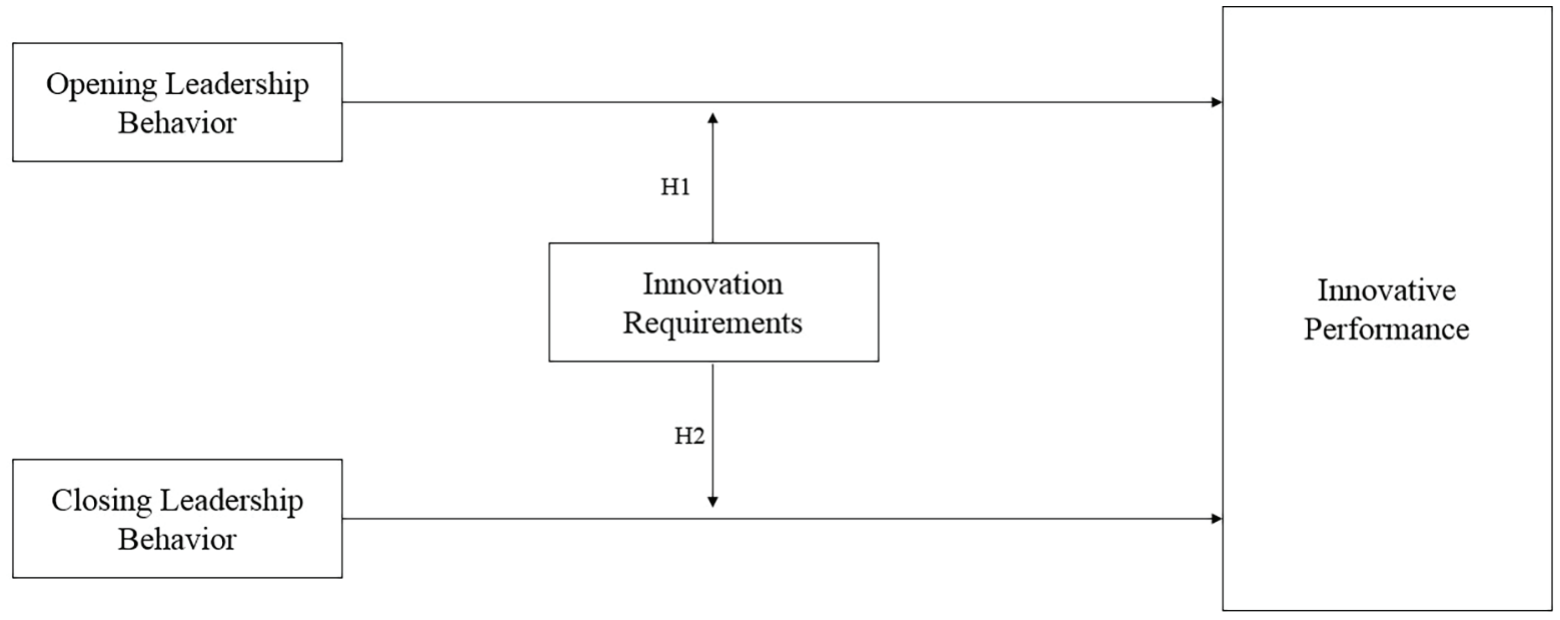
Conceptual model.

Taking these shortcomings into consideration, a central advantage of the present study is the explicit experimental manipulation of both opening and closing leader behavior and innovation requirements, which allows us to draw causal conclusions. Using this more rigorous method, we aim to investigate whether the alignment of leader behaviors with creativity and implementation requirements leads to increased performance. For an illustration of our conceptual model see Figure 1.

Our study contributes to the literature in two ways. First, conceptualizing specific creativity and implementation requirements will change our understanding from general job requirements to be creative or innovative toward a micro-level perspective on situation-specific requirements of creativity and implementation within the innovation process (Rosing et al., 2011, 2018; Shin et al., 2017). Creativity and implementation – even though related – are distinct and the few studies that have examined them separately identified specific antecedents for each aspect (Anderson et al., 2014; Hughes et al., 2018). Therefore, theoretical approaches to the innovation process need to incorporate answers to the question how leaders and employees deal with the different requirements of creativity and implementation. Our within-process perspective therefore considers two types of leader behaviors to address creativity or implementation requirements (Shalley et al., 2004). This perspective is in accordance with contingency theories of leadership and further specifies this approach for the alignment of leader behavior with the different situations within the innovation process (Fiedler, 1971; Peters et al., 1985; Shao et al., 2017). In other words, our study contributes to the leadership literature by specifying and testing a contingency model of leadership that is specific to innovation processes. Importantly, although aligning leader behavior with situational requirements is the core idea of the model of ambidextrous leadership, this theoretical idea has never been tested empirically. Thus, our study provides a crucial rigorous empirical test of a central claim of a contingency model, that is, the model of ambidextrous leadership. Second, we offer a methodological contribution to the literature by expanding leadership and innovation research by drawing causal conclusions regarding the influence of opening and closing leader behaviors on performance.

## Theoretical Background and Hypothesis Development

### The Innovation Process

Innovation is a complex process that includes at least two sub-processes of creativity and implementation (West, 2002b; Amabile and Pratt, 2016; Potočnik and Anderson, 2016). Despite differences between specific innovation models, all models agree that employees need to generate new and useful ideas (Amabile, 1988; Amabile and Pratt, 2016). Consider, for example, an employee who is appointed to create a chat system for employees within the organization to communicate faster. This employee needs to take many different aspects, such as usability or data safety, into account and brainstorm on ideas. However, having many original and useful ideas alone will not be sufficient, as creative ideas also need to be implemented for organizations to actually benefit (Axtell et al., 2000; West, 2002a; Baer, 2012). Looking back on the example, the employee needs to choose solutions for the issues posed concerning the design as well as the data security and produce a prototype to evaluate the existing features. After evaluating and improving the prototype, all aspects need to be implemented in the final product. Only then the employee has successfully finished the assigned task and the organization will be able to use the product. Consequently, in addition to creativity requirements, employees also face implementation requirements when dealing with innovation. In this article, we focus on these two sub-processes and postulate that within the innovation process employees face the requirement to be creative and the requirement to implement (Janssen, 2000; Unsworth et al., 2005; Shin et al., 2017). Importantly, these requirements are inherent to the tasks that individuals need to attend to within the innovation process. Moreover, these requirements need to be differentiated from performance within innovation processes, which is defined as the evaluation of the process outcome (Shalley et al., 2000; Montag et al., 2012; Shin et al., 2017).

Although research has explored the role of requirements as the level of creativity or innovation a job requires in general (Unsworth et al., 2005; Hammond et al., 2011; Robinson-Morral et al., 2013), only very little research has focused on the specific requirements regarding creativity and implementation (Shalley et al., 2004). When creativity is required, novelty (Amabile and Pratt, 2016), divergent insights, and unexpected considerations are needed (Miron-Spektor and Erez, 2017). A creativity requirement is associated with ground-breaking opportunities and risks are often inherent (Andriopoulos and Lewis, 2009). By contrast, implementation relies on efficiency, where discipline, control, and structure are important prerequisites for dealing with the requirement (Andriopoulos and Lewis, 2009; Miron-Spektor et al., 2018). Implementation requires the outcome to be practical, and therefore, the acceptance of boundaries and constraints within the organizational environment is necessary (Miron-Spektor et al., 2011; Miron-Spektor and Erez, 2017). Creativity and idea implementation are related, but are distinct aspects of the innovation process that show very different characteristics (Smith and Tushman, 2005; Bledow et al., 2009; Smith and Lewis, 2011). Indeed, the contradictions inherent to creativity and implementation have been coined as “innovation paradox”, meaning that antecedents that promote creativity are often irrelevant (or even harmful) for implementation and vice versa (Miron-Spektor et al., 2011; Miron-Spektor and Erez, 2017). These contradictions make it necessary to clearly differentiate between creativity and implementation requirements. In sum, both creativity and implementation requirements need to be addressed, and they should be addressed by different leader behaviors (Bledow et al., 2009; Rosing et al., 2011).

### Leadership and Innovation Performance

We propose that leader behaviors can help individuals to deal with creativity and implementation requirements (Rosing et al., 2011; Anderson et al., 2014; Junni et al., 2015). Leadership is a central influence within the innovation process. For instance, providing support for creativity is beneficial (Amabile et al., 2004; Junni et al., 2015; Mainemelis et al., 2015; Hughes et al., 2018). Nonetheless, results concerning the role of traditional leadership models in the innovation process, such as transformational and transactional leadership, are not straightforward (Rosing et al., 2011; Hughes et al., 2018). For example, transformational leadership has been found to both foster and hinder creativity and innovation (Eisenbeiß and Boerner, 2013). In general, relationships of traditional leadership approaches with innovation outcomes vary widely (Rosing et al., 2011; Hughes et al., 2018). We suggest that this variation is due to not explicitly considering the distinct requirements of creativity and implementation that are both inherent to the innovation process (Hunter et al., 2011; Rosing et al., 2011; Gerlach et al., 2020). Thus, these models overlook the necessity to align leader behavior with situational demands of the innovation task, a suggestion that goes back to contingency models of leadership (Fiedler, 1971; Peters et al., 1985) that has not been explicitly included in research on leadership and innovation. The model of ambidextrous leadership takes these shortcomings into account and specifically describes two types of leader behavior that directly correspond to the specific requirements of creativity and implementation: opening and closing leader behavior (Rosing et al., 2011). A study by Zacher et al. (2016) found that opening leader behavior is positively related to creativity-related behaviors, whereas closing leader behavior is positively related to implementation-related behaviors. Moreover, the interaction of opening and closing leader behaviors has been shown to predict overall innovation performance, including creativity and implementation aspects, such that performance is highest when both opening and closing behaviors are high (Zacher and Wilden, 2014; Zacher and Rosing, 2015).

### Alignment of Leader Behaviors and Requirements

Based on propositions of the model of ambidextrous leadership, we examine the differential effects of opening and closing leader behaviors on performance when the moderating influence of innovation requirements is considered. We first suggest that high levels of opening leader behavior will increase performance when creativity requirements are present. Opening leader behavior emphasizes the goal to be creative, which will support individuals to address a creativity requirement (Shalley, 1991; Shalley and Gilson, 2004; Mainemelis et al., 2015). Looking at this in more detail, opening leader behavior provides individuals with room for independent thinking and acting (Rosing et al., 2011). For innovation success, autonomy to decide is important for individuals to find creative solutions (Mumford et al., 2002; Shalley and Gilson, 2004; Hammond et al., 2011) and this is provided by opening leader behavior. Furthermore, to be creative, individuals need to question existing structures and routines (Shalley and Gilson, 2004; Hunter et al., 2007; Miron-Spektor and Erez, 2017). This is encouraged by opening leader behavior (Rosing et al., 2011). Moreover, opening leader behavior provides an environment in which individuals search for new information and knowledge (Rosing et al., 2011; Kremer et al., 2019). This enables creativity, because diversity of knowledge and perspectives will help individuals to access different information and consequently find novel and unusual solutions (Mumford et al., 2002; Shalley and Gilson, 2004; Taylor and Greve, 2006). Moreover, individuals who strive to learn have been shown to perform better in terms of creativity (Gong et al., 2009; Hirst et al., 2009). Such a learning goal orientation is encouraged by opening leader behavior, that is, seeing mistakes as a chance to learn (Rosing et al., 2011). Lastly, a climate for psychological safety is important for employees to unfold their creative potential (Baer and Frese, 2003). This is supported by opening leader behavior, since it provides individuals with safety to voice ideas and take risks (Hunter et al., 2011; Rosing et al., 2011).

In contrast, a high level of opening leader behavior will not be helpful in case of implementation requirements. When implementation is required, employees need to focus on efficiency and quality (Miron et al., 2004; Miron-Spektor et al., 2011). They have to address the implementation in a given environment with constraints and boundaries (Andriopoulos and Lewis, 2009; Miron-Spektor et al., 2011). Opening leader behavior does not focus on these constraints and boundaries, but rather encourages the questioning of existing structures (Rosing et al., 2011). Furthermore, when opening leader behavior is applied, mistakes are seen as a chance to learn (Rosing et al., 2011). This contradicts the notion of implementing a high-quality product in an efficient manner (Miron et al., 2004; Miron-Spektor et al., 2011). Therefore, a high level of opening leader behavior will not support employees when facing these challenges associated with implementation.

*Hypothesis 1*: Innovation requirements moderate the effect of opening leader behavior on performance, such that opening leader behavior has a positive effect on performance in situations that require creativity, but not in situations that require implementation.

We further propose that a high level of closing leader behavior promotes innovation performance, when implementation rather than creativity is required. In general, closing leader behavior emphasizes productivity goals and thus, enables individuals to address implementation requirements (Shalley, 1991; Rosing et al., 2011). More specifically, closing leader behavior is characterized by planning and setting specific goals (Rosing et al., 2011) and thus, shapes an environment in which employees are not distracted by unnecessary activities (Mumford, 2000; Miron-Spektor et al., 2011). In case of implementation requirements, individuals need to focus on the essential goals and be effective (Mumford, 2000; Miron-Spektor et al., 2011). Closing leader behavior includes the monitoring of goal attainment within given deadlines and puts constraints on time as a resource (Rosing et al., 2011). Time constraints are associated with narrow processing and thus, a focus on essential tasks, which is also important for implementation requirements (Mumford, 2000; Amabile et al., 2002; West, 2002b). Moreover, employees are expected to produce high-quality outcomes (Miron et al., 2004). Closing leader behavior encourages individuals to attend to details and avoid mistakes (Rosing et al., 2011). Furthermore, closing leader behavior focuses on existing knowledge and routines, which help individuals to attend to the quality of outcomes, rather than the process (Miron et al., 2004; Jansen et al., 2006; Rosing et al., 2011).

In contrast, the environment created by a high level of closing leader behavior will not be particularly useful in case of creativity requirements. Creativity requirements necessitate engaging in divergent thinking, questioning existing structures, and taking risks to search for different and novel solutions (Andriopoulos and Lewis, 2009; Miron-Spektor and Erez, 2017). As closing leader behavior provides a tight structure by setting deadlines, planning, and monitoring activities (Rosing et al., 2011), this will restrict individuals to focus on specific tasks and activities. This restriction will not be supportive when aiming for a creative solution.

*Hypothesis 2*: Innovation requirements moderate the effect of closing leader behavior on performance, such that closing leader behavior has a positive effect on performance in situations that require implementation, but not in situations that require creativity.

## Materials and Methods

We tested our hypotheses with a 2 × 2 × 2 between-subjects experimental design. In the experiment, we independently manipulated innovation requirements of a task (creativity vs. implementation) and leader behaviors in terms of opening behavior (no opening vs. opening) and closing behavior (no closing vs. closing).

### Sample

Participants were recruited at a German university using a system for psychology students to receive credits for participation in experimental studies. Students from other disciplines were recruited in lectures and through social media and received 10 euros as compensation. We chose a student sample for this study because the processes associated with leader influence on innovation under laboratory conditions should be similar to a controlled setting with an employee sample (Highhouse and Gillespie, 2009). It was our main goal to provide a sufficiently large sample size to ensure statistical power and internal validity (Highhouse, 2009; Highhouse and Gillespie, 2009). Moreover, student samples have often been used in creativity and innovation studies (Franke and Piller, 2004; Baas et al., 2008; Rosing et al., 2018) as well as experimental leadership studies (Stam et al., 2010, 2016). The initial sample size was *N* = 250. Five participants had to be excluded due to computer problems or because they had previously taken part in one of the pre-tests. This led to a final sample size of *N* = 245 with 29–32 participants in each of the eight experimental conditions. Participants were on average 23.35 years old (*SD* = 4.48). There were 66.5% female and 32.7% male participants. Students from a variety of disciplines participated, mostly psychology (49.0%), followed by business studies (12.2%) and engineering (11.0%).

### Procedure

Upon arrival at the laboratory participants were given instructions via an online survey tool (Unipark, Questback GmbH) to assume they were a new employee in the university’s marketing team. They were informed that the team’s goal was to recruit as many high school students as possible for the university and that they would receive their first independent task today. Next, a video message from the team leader was presented. The same male leader in all the videos gave information on how the work in the department was done. He explained the way employees dealt with the tasks in general and emphasized what was important to him. Depending on the leadership condition, the video message contained the manipulation of (high or low levels of) opening and closing leader behaviors. Afterwards, participants received the instruction for a task from the experimenter. The task either required creativity or implementation. Then they were presented with a short, written reminder from the leader. This reminder contained the central points of the leadership manipulation and was also pinned as a paper note to their computer screen. Participants in the experimental group in which neither opening nor closing leader behavior was shown did not receive a reminder, because the video message did not include any specific instructions.

After the reminder, participants received additional information for their respective task (i.e., creativity or implementation) from the experimenter, that is, a written guide that contained information on the standards for the task. Additionally, as the implementation task was done using Microsoft Word, participants in these groups received a set of Microsoft Word tips to even out the differences in skill level. Pre-tests indicated that creativity and implementation tasks required different execution times. Thus, participants had 15 min for the creativity task and 25 min to perform the implementation task. The pretests showed that additional time in the creativity task did not lead to better performance outcomes. Furthermore, they indicated that participants also had sufficient time to complete the implementation task. After the predefined time frame, participants were asked to stop working on the task and the leader’s reminders were removed from the computer screen. Subsequently, participants rated opening and closing leader behaviors of the leader shown in the video as a manipulation check. Finally, participants answered questions concerning control variables: transformational and transactional leadership with respect to the video message as well as demographics.

### Independent Variables

*Leader behaviors* were manipulated in the video messages participants received before task execution. This approach is similar to other experiments manipulating leadership influence in the laboratory (Stam et al., 2010, 2016; Jacquart and Antonakis, 2015). The same male leader was presented in all videos. Aspects of *opening leader behavior*, such as questioning existing rules and routines, were either part of the video message (opening) or not (no opening). Likewise, *closing leader behavior*, such as the instruction that it is necessary to follow existing routines and guidelines were either shown in the video (closing) or not (no closing). This resulted in a total of four different videos: neither opening nor closing leader behavior (no leadership control group), solely opening, solely closing, as well as both opening and closing leader behaviors. The control group video contained information on the team and the tasks they generally do and thus, no specific instructions concerning the task at hand were given. A detailed list of opening and closing leader behaviors is shown in Table 1. In addition, we actively included aspects of transformational leadership (such as communicating a vision) in all conditions to keep these aspects constant across conditions. For the full-length scripts of the leadership manipulation refer to the Supplemental Material (https://osf.io/rnpj4/?view_only=c87994dbbdc145e7bf71b3339b16ca13).

**Table 1:** Manipulation of opening and closing leader behaviors.

Opening leader behavior	Closing leader behavior
Unconventional ideas and creativity	Productivity and efficient implementation
Try out different and new things	Work per predefined plans, tasks, and rules
Be original	Be attentive to details
Deal with different positions and opinions	Be accurate
Different ways to reach goal	Systematic and goal-oriented work
Mistakes as chance to learn	Resort to proven routines
Take risks	Avoid all mistakes
New ideas detached from old knowledge and standards	Be efficient (fast and free of mistakes)

*Innovation requirements* were manipulated by presenting participants with either a creativity or an implementation task. For the purpose of this research, to allow a comparison between the two requirements, creativity and implementation requirements were manipulated separately as a dichotomous variable for innovation requirements. All materials concerning the tasks are provided in the Supplementary Material. On the one hand, the *creativity task* asked participants to come up with ideas for the marketing of the university (see Supplementary Material). This task is similar to other creativity tasks (De Dreu et al., 2008; Bledow et al., 2013). The written guide for this task contained categories and examples for ideas as well as a flyer for a marketing instrument already implemented at the university. On the other hand, the *implementation task* asked participants to finalize a recruiting brochure about the university (see Supplementary Material). We included mistakes in terms of grammar, punctuation, and formatting, which participants were supposed to correct. We also provided additional material such as pictures to ensure that participants could redesign the brochure. The written guide for the implementation task informed participants about the corporate design and standards concerning formatting and phrasing. The guide also included a flyer as an example for a marketing instrument. The tasks showed a clear requirement of either creativity or implementation as task instructions pointed out that the outcome would be judged according to the requirement. Nonetheless, for a more realistic approach creativity and implementation tasks gave participants options to show different behaviors. For instance, in the creativity task participants could draw on existing knowledge and identify ideas closely related to the guide, or discover new aspects and come up with original ideas that were not associated with those in the guide. For the implementation task, participants could rely on the guide and correct the mistakes in the brochure, or they had the opportunity to redesign the brochure in terms of new pictures or paragraphs.

### Dependent Variable

The dependent variable *performance* was measured separately for the two different tasks. The task instructions for both tasks provided a clear goal for the task in line with the requirement and accordingly, the performance for each can only be evaluated in line with the respective instruction (Nusbaum et al., 2014; Reiter-Palmon et al., 2019). Thus, in the creativity task, we evaluated creativity as an outcome and in the implementation task, we assessed implementation.

According to the definition of creativity as novel ideas (Amabile, 1988; Amabile and Pratt, 2016), *creativity performance* was measured as a percentage of the number of new ideas compared to the number of total ideas generated by each participant (Hagtvedt et al., 2016). In line with existing research on brainstorming tasks, we used a rater based assessment for this purpose (Barbot et al., 2019; Reiter-Palmon et al., 2019). First, a trained research assistant (coder one) counted all the ideas developed during the creativity task for each participant. Initial interrater reliability calculated based on the intraclass correlation coefficient (ICC; Shrout and Fleiss, 1979) with codings from coder one and the first author was based on *n* = 10 answers and showed a very good agreement, *ICC* = 0.99. Subsequently, coder one counted the ideas for all answers. As the task was intended to yield new ideas compared to ideas already in the guide provided to participants (Hagtvedt et al., 2016), another trained research assistant (coder two) identified those ideas from all the ideas counted that were new. Initial interrater reliability with codings from coder two and the first author was calculated based on *n* = 20 answers and showed satisfactory agreement, *ICC* = 0.98. Afterwards, coder two coded the other answers in terms of new ideas. Finally, we calculated the proportion of new ideas on all ideas the participant had generated and used this percentage as the dependent variable measuring creativity performance.

Implementation has been defined as the reliable and efficient handling of a task resulting in a high-quality product (Miron et al., 2004; Miron-Spektor et al., 2011). Therefore, *implementation performance* was assessed as the number of mistakes in the brochure that were corrected by participants within the given time frame. Participants who focused on an efficient way to improve the brochure would first attend to the most necessary aspects, such as the correction of obvious mistakes instead of redesigning the brochure. Initial interrater reliability based on Cohen’s Kappa (Cohen, 1960) was established with the ratings of coder one and the first author. Fifty-seven mistakes were coded in terms of corrected vs. not corrected and, on average, the agreement based on *n* = 20 brochures was very good, *κ* = 0.90. Subsequently, coder one coded all remaining brochures in terms of corrected mistakes. Since it would not be considered high implementation performance to simply “correct” existing mistakes by removing text and adding new text with more mistakes, we further counted the additional mistakes, such as spelling, punctuation, or introduction of new colors different from the corporate design. Initial interrater reliability based on ICC was established with the ratings of coder one and the first author based on *n* = 20 brochures, *ICC* = 0.88. Afterwards, coder one counted all additional mistakes in the remaining brochures. Finally, the number of additional mistakes was subtracted from the number of corrected mistakes. We then calculated this number of mistakes per minute, a measure of efficiency, as the dependent variable representing implementation performance.

As our hypotheses are moderation hypotheses that required us to compare the impact of leader behavior on performance across tasks, we needed to create a single performance measure from the two creativity and implementation performance measures. In order to do so, we z-standardized the creativity and implementation performance variables to create the same metric for both measures. That is, after z-standardization, a value of “1” represents a comparable performance in both tasks as performance was one standard deviation above the mean within the respective tasks. The z-standardized values from the two performance measures were then merged into one performance measure. Each participant, thus, received one z-standardized value for performance resulting from the score of the task they had completed.

### Measures

#### Leader Behaviors

Opening and closing leader behaviors were assessed by participants using the scale developed by Zacher and Rosing (2015). A German version consisting of seven items for each opening and closing leader behaviors was provided by the authors. Items were rated on a five-point scale ranging from 1 = not at all to 5 = very strongly. An example for opening leader behavior was “My leader gives me the possibility to think and act independently” and the scale showed an excellent reliability, *α* = 0.92. An example for closing leader behavior was “My leader sanctions mistakes.” The reliability was also very good, *α* = 0.85.

#### Control Variables

We controlled for transformational and transactional leadership because these leadership styles have frequently been investigated in the context of innovation (Rosing et al., 2011; Hughes et al., 2018). For instance, the influence of transformational and transactional leadership on creativity (Henker et al., 2015) or innovation (Waldman and Bass, 1991; Jansen et al., 2008) has been analyzed. Furthermore, research points to some theoretical overlaps of transformational and transactional leadership with ambidextrous leadership (Rosing et al., 2011). To measure transformational and transactional leadership, we used the Multifactor Leadership Questionnaire (MLQ Form 5x Short) provided in a German translation (Avolio et al., 1999; Felfe, 2006). Transformational leadership was measured with 19 items, as one item was excluded in the German version (Felfe, 2006). An example item was “My leader talks optimistically about the future,” and reliability was excellent, *α* = 0.93. Transactional leadership was measured using seven items, as one item was excluded in the German version (Felfe, 2006). “My leader is mainly concerned with mistakes and complaints,” was an example for this scale and the reliability was satisfactory, *α* = 0.68.

## Results

Intercorrelations of all study variables and the experimental conditions as well as means and standard deviations are displayed in Table 2. As expected, there were no significant correlations between performance and the independent variables. Transformational leadership showed a positive correlation with opening leader behavior (*r* = 0.41, *p* < 0.001) and transactional leadership with closing leader behavior (*r* = 0.34, *p* < 0.001). This is not surprising, as Rosing et al. (2011) pointed out that these leadership constructs are distinct but related. Nonetheless, we controlled for transformational and transactional leadership ratings.

**Table 2:** Intercorrelations of experimental conditions and variables.

	*x-*	*SD*	1	2	3	4	5	6
1. Opening leader behavior (IV)^a^								
2. Closing leader behavior (IV)^b^			−0.00					
3. Innovation requirements (IV)^c^			−0.01	−0.02				
4. Performance (DV)			0.06	0.09	0.01			
5. Transformational leadership	3.28	0.71	0.41^***^	−0.20^**^	0.02	−0.10		
6. Transactional leadership	3.27	0.62	−0.31^***^	0.34^***^	0.05	−0.11	0.07	

*N* = 245. IV = independent variable; DV = dependent variable.

a 0 = no opening leader behavior; 1 = opening leader behavior.

b 0 = no closing leader behavior; 1 = closing leader behavior.

c 0 = creativity requirement; 1 = implementation requirement.

** *p* < 0.01,

*** *p* < 0.001.

### Manipulation Check

We conducted two regression analyses to investigate whether the manipulation of leader behavior was successful. These analyses controlled for transformational and transactional leadership. First, the manipulation of opening leader behavior in the video message had a significant positive effect on ratings of opening leadership (*B* = 1.01, *SE* = 0.08, *p* < 0.001), whereas the manipulation of closing leader behavior had a significant negative effect on ratings of opening leadership (*B* = −0.42, *SE* = 0.078, *p* < 0.001). When opening leader behavior was included in the video, participants rated opening leadership higher (*x-* = 4.21, *SD* = 0.66) compared to when opening leader behavior was not included (*x-* = 2.74, *SD* = 0.83). Second, opening leader behavior had a significant negative effect on the ratings of closing leadership (*B* = −0.58, *SE* = 0.09, *p* < 0.001), whereas closing leader behavior had a positive effect on ratings of closing leadership (*B* = 0.46, *SE* = 0.08, *p* < 0.001). When closing leader behavior was included in the video message, participants rated closing leadership higher (*x-* = 3.45, *SD* = 0.74) compared to when closing leader behavior was not included (*x-* = 2.71, *SD* = 0.78). Therefore, we can conclude that the manipulations of opening and closing leader behavior in the video messages were successful.

### Hypothesis Tests: Leader Behaviors and Requirements

**Figure 2:**
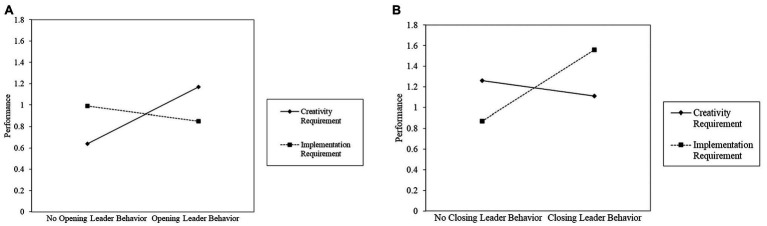
Interaction effects of (A) opening leader behavior and (B) closing leader behavior with requirements on performance.

Hypothesis 1 states that opening leader behavior fosters performance when creativity requirements are present. Hypothesis 2 postulates that closing leader behavior leads to better performance in case of implementation requirements. Hierarchical regression was used to analyze the effects of two-way interactions between leader behaviors and requirements on performance (Aguinis et al., 2005, 2017)^1^. Results are reported in Table 3. First, the control variables transformational and transactional leadership were included into the regression equation in Step 1 but did not have significant effects. In Step 2, the independent variables opening and closing leader behavior as well as the requirements were added to the equation. No significant influences of the main effects were found. In Step 3, all two-way interactions between opening leader behavior, closing leader behavior, and requirements were included; a significant increase in *R* ^2^ was observed. The interaction of opening leader behavior and requirements was significant (*B* = −0.67, *SE* = 0.25, *p* = 0.007). This interaction effect is displayed in Figure 2A. As expected, simple slope analysis revealed that the effect of opening leader behavior on performance was significant and positive when the task required creativity (*B* = 0.54, *SE* = 0.19, *p* = 0.005). The effect was not significant in case of implementation requirements (*B* = −0.13, *SE* = 0.19, *p* = 0.50). These results support Hypothesis 1.

**Table 3:** Hierarchical regression of performance on leader behavior and requirements.

	Model 1	Model 2	Model 3	Model 4
STEP 1				
Transformational leadership	−0.09	−0.10	−0.08	−0.08
Transactional leadership	−0.10	−0.11	−0.15^*^	−0.15^*^
STEP 2				
Opening leader behavior^a^		0.08	0.25^*^	0.23
Closing leader behavior^b^		0.11	−0.06	−0.08
Requirements^c^		0.01	−0.02	−0.04
STEP 3				
Opening^a^ × Closing^b^			−0.04	−0.00
Opening^a^ × Requirements^c^			−0.29^**^	−0.26
Closing^b^ × Requirements^c^			0.36^**^	0.39^*^
STEP 4				
Opening^a^ × Closing^b^ × Requirements^c^				−0.05					
Δ *R* ^2^	0.02	0.02	0.07	0.00
*R* ^2^	0.02	0.04	0.11	0.11
F-Change	2.36	1.44	6.24^***^	0.10

*N* = 245.

a 0 = no opening leader behavior; 1 = opening leader behavior.

b 0 = no closing leader behavior; 1 = closing leader behavior.

c 0 = creativity requirement; 1 = implementation requirement.

Standardized regression coefficients (*b*) are reported. Dependent variable = Performance.

* *p* < 0.05,

** *p* < 0.01,

*** *p* < 0.001.

Moreover, the interaction of closing leader behavior and requirements showed a positive effect on performance (*B* = 0.82, *SE* = 0.25, *p* = 0.001). The interaction is displayed in Figure 2B. Simple slope analysis revealed a positive effect of closing leader behavior on performance in case of implementation requirements (*B* = 0.69, *SE* = 0.19, *p* < 0.001). The effect was not significant when the task required creativity (*B* = −0.15, *SE* = 0.18, *p* = 0.40). These findings provide support for Hypothesis 2.

In the final step of the regression analysis, the three-way interaction of all independent variables was added, but did not show a significant effect. This points to the fact that showing opening and closing leader behaviors simultaneously does not influence the performance outcome.

## Discussion

Creativity and implementation requirements are highly relevant within innovation processes, as they determine the behavior that is effective for leaders in a given situation. From our results, we can conclude that opening leader behavior has a positive influence on performance when creativity is required (Hypothesis 1), whereas closing leader behavior leads to better performance in case of implementation requirements (Hypothesis 2). In contrast, none of the leader behaviors studied had a general effect on performance, that is, independent of the requirements of the tasks. These results suggest that different leader behaviors are only effective depending on the presence of either creativity or implementation requirements, and therefore, these situational demands are highly relevant for innovation success (Shalley et al., 2004; Jankowska et al., 2018). These findings are in line with the propositions of the ambidextrous leadership model (Rosing et al., 2011). Our results further support the model’s propositions, because the effects of opening and closing leader behaviors emerged, while controlling for the traditional approaches of transformational and transactional leadership.

Through a rigid ambidexterity lens, one could assume that showing both opening and closing leader behaviors at the same time would further increase performance outcomes (He and Wong, 2004; O᾽Reilly and Tushman, 2013). In contrast, our results did not reveal a significant effect of the two-way interaction of opening and closing leader behavior. This means that the video including both opening and closing statements did not further improve innovation performance. This is not surprising when considering that only one requirement of either creativity or implementation was presented and not both at the same time. In line with these results, Shalley (1991) found that applying two different goals does not influence performance, when one of the goals is aligned with the task requirements. Therefore, when a clear requirement of either creativity or implementation is present, the simultaneous application of opening and closing leader behavior did not have an effect. In addition, we did not find a three-way interaction including task requirements on performance outcomes. Thus, the conditional effect of opening leader behavior on performance was not dependent on closing leader behavior or vice versa. This again can be interpreted as such that the effect of one type of leader behavior on one type of performance was neither improved nor hindered by the presence or absence of the other type of leader behavior.

### Theoretical and Methodological Contributions

This research makes two key contributions that yield new insights concerning the influences of leadership on innovation performance. First, our study examined the impact of situational requirements within the innovation process in detail (Shalley et al., 2004). Previous research has either investigated jobs that require innovation as a whole (Shin et al., 2017) or has considered only creativity requirements (Shalley et al., 2000; Unsworth et al., 2005). Such a broad perspective, however, makes it difficult to understand the role of aligning leader behavior to innovation requirements comprehensively. Until now, researchers have neglected the different requirements of creativity and idea implementation within the innovation process (Janssen, 2000; Unsworth et al., 2005; Shin et al., 2017). Although there seems to be an implicit agreement in innovation research that innovation includes both creativity and implementation and that these two sub-processes are very different in nature, the distinction between creativity and implementation and their specific requirements have mostly been disregarded in empirical research (Baer, 2012; Peralta et al., 2015). From our theoretical viewpoint – and the differential effects found in our experiment underline this argument – it is important to consider both requirements to understand leadership for innovation, as they establish which leader behaviors are effective in a given situation (Rosing et al., 2011). Using a contingency approach to leadership (Fiedler, 1971; Peters et al., 1985), we, thus, contribute by specifying and studying specific leader-situation contingencies that are relevant for innovation. Methodologically, we contribute to existing research in this area by manipulating creativity and implementation requirements in an experimental setting. This allows us to draw stronger causal conclusions concerning the differential influences of these requirements, compared to existing correlational studies (Unsworth et al., 2005; Zacher et al., 2016; Shin et al., 2017). Regarding the results of this study, it enables a rigorous empirical test of ambidextrous leadership as a contingency model of leadership for innovation processes (Rosing et al., 2011).

Second, we studied the influence of specific leader behaviors on performance in two tasks under controlled conditions. Previous research on leadership in innovation processes points to the need for more objective measures as well as the experimental – and thus causal – analysis of the proposed models, including ambidextrous leadership (Zacher and Wilden, 2014; Zacher and Rosing, 2015; Zacher et al., 2016; Hughes et al., 2018). This need has been successfully addressed in our study and shows important additional support for the effectiveness of opening and closing leader behaviors with respect to the innovation process (Rosing et al., 2011). Experiments have many advantages compared to field studies, particularly that it is easier to control influences that are not of central importance to the research questions under investigation (Antonakis et al., 2010, 2014; Antonakis, 2017; Hughes et al., 2018). Specifically, in this experiment, we controlled for additional situational cues such as performance information that could change the perception of leaders (Lord et al., 1978; Wang et al., 2019). Therefore, causal conclusions can be drawn from our observed results, and we can be confident that leader behavior did influence performance and not vice versa. One additional very important aspect in this regard is the objective assessment of performance. In field studies, innovation outcomes are usually rated either by the employees themselves (e.g., Axtell et al., 2000; Zacher and Wilden, 2014; Zacher et al., 2016) or their supervisors (e.g., Janssen, 2000; Shalley et al., 2004). These two methods have a number of limitations (Hülsheger et al., 2009; Barbot et al., 2019). Most importantly, self-ratings of innovation performance are correlated with motivation and self-efficacy for innovation and, therefore, their validity can be questioned (Reiter-Palmon et al., 2012, 2019; Barbot et al., 2019). Within our experimental setting, outcome evaluation was more objective since it was rated by independent observers after the task completion (Reiter-Palmon et al., 2019). These raters were blind to the experimental manipulations and the outcomes for the tasks. This further adds to the strength and robustness of the reported analyses.

### Limitations and Future Research

As with all research, this study has some limitations. First, like in other laboratory studies, the generalizability of our results is limited (Hoyle et al., 2008). The tasks for creativity and implementation requirements separated the two aspects, which is not common to innovation processes in an organizational setting (Schroeder et al., 1989; Bledow et al., 2009). Usually, employees address the different requirements more flexibly, and thus, the separation seems artificial. Nonetheless, a separation of creativity and implementation was central to the aim of this study. Research shows promising results for the effectiveness of ambidextrous leadership behaviors in the field (Zacher and Wilden, 2014; Zacher and Rosing, 2015; Zacher et al., 2016). Thus, in order to provide causal support for the boundary conditions of the effects of opening and closing leader behaviors, the separate manipulation of creativity and implementation requirements was necessary. Another deficiency lies in the student sample, which might also limit the generalizability of results. However, there is almost no empirical evidence showing that student samples actually differ from workplace samples, since most studies found similar effects for both samples (Highhouse and Gillespie, 2009). Furthermore, experiments mainly aim to provide support for causes and effects of theoretical constructs (Highhouse, 2009; Antonakis et al., 2010, 2014). Therefore, the first objective is to focus on manipulation strength and sample size to receive sufficient power to detect causal effects (Highhouse, 2009; Highhouse and Gillespie, 2009). The main goal of our study was to provide theoretical generalizability for the model of ambidextrous leadership (Highhouse and Gillespie, 2009). Lastly, the conditions of the experiment did not represent an actual workplace situation as students were in a laboratory and only imagined to be employees in the university’s marketing team. However, it was not the goal of the experiment to provide external validity in terms of workplace conditions but rather to keep these influences constant across all experimental groups to enable causal conclusions concerning the influence of leader behaviors (Highhouse, 2009; Antonakis, 2017). Nonetheless, further research should investigate the postulated relationships in a field setting to provide further support in terms of external validity for the ambidextrous leadership model.

Second, as we focused on comparing creativity and implementation requirements as boundary conditions, our study did not consider the dynamic interplay or temporal pattern of creativity and idea implementation (Rosing et al., 2018). However, the influence of innovation requirements within the innovation process will be especially interesting when considering the flexible interplay of both requirements (Schroeder et al., 1989; Cheng and van de Ven, 1996). Researchers have stated that creativity and implementation do not follow a linear temporal pattern and empirical results support this assumption (Schroeder et al., 1989; Rosing and Zacher, 2017; Rosing et al., 2018). It follows that individuals need to change their behaviors regularly to address the changing requirements, resolve associated tensions within the innovation process, and act ambidextrously (Miron-Spektor and Erez, 2017; Rosing and Zacher, 2017; Miron-Spektor et al., 2018). Based on this research, the ambidextrous leadership model also suggests that leaders need to apply opening and closing leader behavior in a temporally flexible manner contingent on the requirements (Rosing et al., 2011). With this temporal flexibility, ambidextrous leader behavior should support employees to resolve the paradox of creativity and implementation (Rosing et al., 2011; Smith and Lewis, 2011; Miron-Spektor et al., 2018). This experiment was conducted with an explicit separation of creativity and implementation because we aimed to compare the influences of behaviors under the different requirements. As pointed out in the section above, this gives us a first insight that using adequate behaviors will be helpful to address the different requirements. Nonetheless, over time both requirements will be present within the innovation process and this needs to be addressed in future studies. Thus, it will be necessary to address the flexible interplay of opening and closing leader behaviors – actual ambidextrous leadership – with the respective requirements in future experimental as well as field studies (Rosing et al., 2011). These studies would advance a more complete understanding of the influence of leader behaviors within the innovation process.

Finally, future research needs to focus more specifically on antecedents and consequences of opening and closing leader behaviors in comparison to other leadership approaches. For instance, even though studies, like the present one, show that ambidextrous leadership explain variance above transformational and transactional leadership (Zacher and Rosing, 2015; Zacher et al., 2016; Gerlach et al., 2020), high correlations among the constructs point to a relatedness that needs to be further explored (Rosing et al., 2011). Specifically, future research needs to examine the nomological net of ambidextrous leadership in field studies. To date, studies examining the relative predictive validity of different leadership approaches (including ambidextrous leadership) are rare (Hughes et al., 2018; Gerlach et al., 2020). However, these studies are important to differentiate which leadership models and behaviors are most effective (Hughes et al., 2018). This is especially relevant for the complex innovation process (Rosing et al., 2011; Hughes et al., 2018; Gerlach et al., 2020).

### Practical Implications and Conclusion

Results of this study clearly point to the importance of situational demands of creativity and implementation in innovation processes. Innovation processes will be more successful if both requirements are considered and addressed by leaders (Bledow et al., 2009). Leaders who show opening leader behavior set a frame that enables followers to address creativity requirements, whereas leaders showing closing leader behavior will help followers when meeting implementation requirements (Rosing et al., 2011). Thus, paying attention to creativity and implementation requirements within the innovation process will contribute to better innovation outcomes.

More research with respect to leadership and innovation needs to be conducted, as we currently cannot draw causal conclusions regarding the flexible interplay and integration of leader behaviors. Nonetheless, our research contributes to the literature in that it points to the importance of a more detailed review of situational aspects such as innovation requirements. Prior literature shows that creativity and implementation are relevant aspects of the innovation process (Schroeder et al., 1989; Rosing et al., 2018). Our study adds to this understanding as we considered creativity and implementation requirements as essential part of the innovation process and provide evidence that differential leader behaviors are necessary to adequately address creativity and implementation within this process. This is a promising avenue for future research as a more micro-level perspective on the innovation process will allow us to draw conclusions on the conditions under which leader behaviors will lead to successful innovation processes.

## Data Availability Statement

The datasets generated for this study are available on request to the corresponding author.

## Ethics Statement

Ethical review and approval was not required for the study on human participants in accordance with the local legislation and institutional requirements. Written informed consent for participation was not required for this study in accordance with the national legislation and the institutional requirements.

## Author Contributions

FG: conception and design, data analysis and interpretation, drafting of the article, and critical revision of the article. KH: conception and design, data preparation, and drafting of the article. KR: conception and design, helped with data analysis and interpretation, and critical revision of the article. HZ: data analysis and interpretation and critical revision of the article. All authors contributed to the article and approved the submitted version.

### Conflict of Interest

The authors declare that the research was conducted in the absence of any commercial or financial relationships that could be construed as a potential conflict of interest.

## Footnotes

^1^Before analyzing the data using the combined performance measure, we analyzed the effects of opening and closing leader behavior on performance for each task separately. Thus, we regressed creativity and implementation performance on opening and closing leader behavior while controlling for transformational and transactional leadership. As expected, these analyses revealed a significant positive effect of opening leader behavior on creativity performance (*B* = 0.44, *SE* = 0.20, *p* = 0.030) and a significant positive effect of closing leader behavior on implementation performance (*B* = 0.60, *SE* = 0.21, *p* = 0.006).

## Supplementary Material

The Supplementary Material for this article can be found online at: https://www.frontiersin.org/articles/10.3389/fpsyg.2020.01332/full#supplementary-material (also available at https://osf.io/rnpj4/?view_only=c87994dbbdc145e7bf71b3339b16ca13).
