# Supplementary material for: Aligning Leader Behaviors With Innovation Requirements Improves Performance: An Experimental Study
Source: Front Psychol. 2020 Jul 7;11:1332. doi: 10.3389/fpsyg.2020.01332 (PMC7358614; doi:10.3389/fpsyg.2020.01332)

Creativity Task Instructions

Please imagine you would be an employee of the marketing department of your university. The war for talent is already starting at the universities. Therefore, it is especially important to recruit students for your university. You just started the position in the marketing team and now you will receive you first independent task.

The goal of your team in the marketing department of the university is to address as many students as possible and to recruit them for your university. Now, as one of your first tasks, you are asked to generate as many ideas as possible on how students can be addressed by your university. Please enter your ideas in the text box on the next page. To give you an idea of what is being done in the field of university marketing, you will get a few details that can serve as a guide.

You have 15 minutes to complete this task. Before you start, the experimenter will provide you with the additional information.

Please consider as many ideas as possible on how students can be approached by your university. Please simply enter them in the text box below.

You have 15 minutes to complete this task. The experiment management will let you know when the time is up, and you can continue with the next task.

Guide for the Creativity Task

To help you to perform best at the task, here are some details concerning previous international university marketing activities aimed at attracting foreign students:

Print Media

- Brochures or leaflets on the university and study programs in German and English; Distribution of information material for display at institutions abroad

Digital Media

- e-mail counseling for foreign students; University web pages or parts of university web pages in English; PowerPoint presentations for information on Erasmus programs

Events

- visit of partner universities; visiting other foreign universities (beyond partner universities); Participation in fairs on topics such as "Semester Abroad" and "Erasmus"

Radio and Movies

- Image films about the International Office; Radio contributions that report on and inform about studying abroad

Here's an example of what international university marketing advertising can look like.

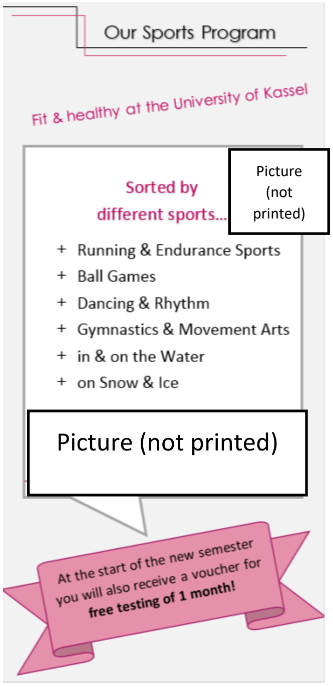

Supplement: Supplementary file 1 [file Data_Sheet_1.PDF]
