# Supplementary material for: Aligning Leader Behaviors With Innovation Requirements Improves Performance: An Experimental Study
Source: Front Psychol. 2020 Jul 7;11:1332. doi: 10.3389/fpsyg.2020.01332 (PMC7358614; doi:10.3389/fpsyg.2020.01332)

Implementation Task Instruction

Please imagine you would be an employee of the marketing department of your university. The war for talent is already starting at the universities. Therefore, it is especially important to recruit students for your university. You just started the position in the marketing team and now you will receive you first independent task.

The goal of your team in the marketing department of the university is to address as many students as possible and to recruit them for your university. Now, as one of your first tasks, you are asked to complete the brochure for students. For this task an old version will be provided. Your predecessor started this task, but unfortunately did not have the opportunity to finalise the brochure. Now it is your job to improve and correct the existing brochure to finalise this task. Additional materials are also available. The experimenter will show you shortly where to find them. If you want to change something but do not know how to do it in Word, just write it down in the boxes provided on the last page in the document. To give you an idea on how we usually design these brochures, you'll get a few details that can serve as a guide. In addition, we have put together some tips for you that can help you while editing the brochure in the Word document.

You have 25 minutes to complete the task. If you are done earlier, just let the experimenter know. Before you start, the experimenter will provide you with the additional information.

Guide for the Implementation Task

To help you perform best at the task, here are some details concerning our standards in terms of graphic and formal aspects that should be considered in the implementation:

1. Uniform colour scheme of a graphic template
    - The colours should be similar within each product. This means that the established colour palette of the corporate design should not be supplemented by new shades of colour.

2. Error-free spelling, grammar and comma
   - Spelling, grammar or punctuation errors should not occur in the final products.
3. Uniform formatting of pictures and readability of texts
   - The images and graphic elements used should be arranged in a way that the text is easy to read and overall formatted in a consistent manner.
4. Uniform formatting of the font
   - The font should be kept consistent within a product. The headline, subheadings and text can be formatted differently. However, all headings (e.g.) or all text elements should be the same.
5. Plausibility of content
   - The content of the product should be varied interestingly, but still plausible in content. All content aspects should be presented realistically.

Here's an example of what international university marketing advertising can look like.

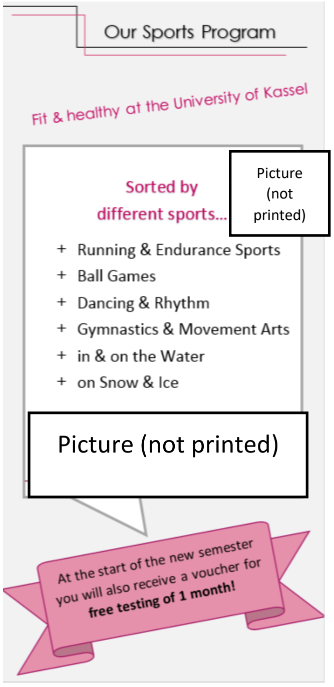

Supplement: Supplementary file 2 [file Data_Sheet_2.PDF]
