# Supplementary material for: Aligning Leader Behaviors With Innovation Requirements Improves Performance: An Experimental Study
Source: Front Psychol. 2020 Jul 7;11:1332. doi: 10.3389/fpsyg.2020.01332 (PMC7358614; doi:10.3389/fpsyg.2020.01332)
Supplement: Supplementary file 3 [file Data_Sheet_3.PDF]

Leadership Manipulation Introduction

Hello,

You do not know me yet and I'd like to briefly introduce myself: My name is Thomas Meier. I am the head of university marketing at the University of XYZ and thus your supervisor. Welcome to my team!

I will give you a task which will be presented to you in detail soon. In general, it's about attracting as many students as possible to our university.

Leadership Manipulation Text Modules

No Leadership

Our department consists of eight staff members, each dealing with different aspects of university marketing, an assistant and myself. Specifically, we work on the development of marketing tools for our university. We do this by differentiating between the various target groups: high school students, university students, academic staff and professors. For each target group, two employees work together, with one colleague who serves the target group "professors" only being in the office half-days. This is the first information I can give you about the team and our work. Regarding your task, you will get more detailed instructions and information in a moment.

Opening Leadership Behaviour

To me, it is especially important that we create high quality products to convince many students to come to our university. I will now give you initial ideas on how we work in our department to achieve this goal.

First of all, our marketing department represents creativity and unconventional ideas. It is important to me that we all work together to act on this maxim. To me, it is important that you try different things and think outside the box, too. Be original and give options a try. I expect you to deal with different opinions and new perspectives. In addition, I value novelties, so that we create high quality products in the end!

The tasks you are going to work on can be done in different ways. Since there are several ways to accomplish this goal, we have very few guidelines for how you handle the tasks. It is important that you experiment with different ideas to improve the quality of the products. In my experience, it is necessary that you try out new ways of thinking and new options. Mistakes may happen in this process. Consider mistakes as a possibility you can use to achieve a better result.

In our department it is necessary to take risks. I expect you to try out different options. In my experience, this energy is well invested. It is important to me, that we are free from old regulations and ideas, to develop original and different products in the end.

Closing Leadership Behaviour

To me, it is especially important that we create high quality products to convince many students to come to our university. I will now give you initial ideas on how we work in our department to achieve this goal.

First of all, our marketing department represents productivity and efficient implementations. It is important to me that we all work together to act on this maxim. To me, it is important that you stick to the given plans, tasks and rules. Be sensitive to the details and work with care. I expect you to proceed in a systematic and goal-oriented manner. In addition, I value the adherence to rules and regulations, so that we create high quality products in the end! The tasks you are going to work on can best be done if you get to know the details of the task at first. It is important that you consider these while carrying out the task to improve the quality of our products. In my experience, it is necessary that you rely on well-tried patterns and routines. This is the best way to avoid mistakes. Freedom from error is important to achieve the best possible result.

In our department it is necessary to work efficiently. Since we have a lot of work, it is important that you get to a faultless result as soon as possible. Focus your power on the

essentials. In my experience, this energy is well invested. It is important to me that we develop neat and error-free products in a timely and accurate way in the end.

Opening & Closing Leadership Behaviour:

To me, it is especially important that we create high quality products to convince many students to come to our university. I will now give you initial ideas on how we work in our department to achieve this goal.

First of all, our department of university marketing represents creativity and efficient implementation. It is important to me that we all work together to act on this maxim. To me, it is important that you stick to the given plans and rules. At the same time, it is important that you try different options and think outside the box, too. Be original and give something a try. But be sensitive to the details and work with care, too. I expect you to both proceed in a systematic and goal-oriented manner as well as deal with different opinions and new perspectives. On the one hand I value novelties, but on the other hand, the adherence to rules and regulations is important in order to create high quality products in the end!

The tasks we have here can best be done if you get to know the details of the task first and consider these while carrying it out. Different ways can lead to this goal. It is important that you experiment with different ideas, but also consider the instruction in the task while working to improve the quality of the products.

In my experience, you should both try new thinking patterns and new options as well as rely on well-tried routines. Mistakes should be avoided and above all not repeated. If mistakes occur, understand them as a possibility you can use to achieve a better result. In our department it is necessary to take risks and at the same time work efficiently, too. Try to test different options but still try to achieve an error-free result as fast as possible. For this endeavour, it is wise to focus your power on the essentials at some point. It is important to me that we develop original and error-free products in a time-efficient way and free of old regulations and ideas in the end.

General Farewell

I am confident that with our products we will be able to address students more in order to recruit them for our university. Every employee is important and I personally value that everyone can contribute their individual skills and talents. Your job is very interesting for our team and the university marketing. If we can achieve our goals and recruit more students through the products, I am very satisfied with our work.

Now, I wish you a successful day at work and good luck with the first task!
