# Supplementary material for: Aligning Leader Behaviors With Innovation Requirements Improves Performance: An Experimental Study
Source: Front Psychol. 2020 Jul 7;11:1332. doi: 10.3389/fpsyg.2020.01332 (PMC7358614; doi:10.3389/fpsyg.2020.01332)
Supplement: Supplementary file 4 [file Data_Sheet_4.PDF]

University Ticket

Picture

(not printed)

Did you know that you as a student of the University of XYZ

benefit from our cultural ticket?

This allows you to enjoy perks or even in free entry to some cultural highlights here in Kassel and discover youre new home. The following institutions participate, Among Others:

- **theater**
- **comedy**
- **museum**
- **event locations**
- **…und manie more**

The city is looking forward to seeing you!

Uniparties

Let´s Celebrate!

Picture

(not printed)

"Welcome-Paty"

In the first week of the semester, it's time to say'Welcome dear Freshman! 'and the old primary school in XYZ will be converted into the ultimate venue! With hot beats and trendy DJ's from the region you can dance on several flooors, celebrate the start of University and get to know new fellow studentis Drinks for a small budget will also be waiting for you.

About all other parties, you will always be informed on the campus or through our **Facebookgroup**. Bye for now)

Picture

(not printed)

U N I V E R S I T Ä T

After high school is before college life

Here you will find current benefits oft he University of XYZ:

- University sports: Stay in motion!
- Workshops: Train your Soft Skils!
- Semester abroad: Around The World!
- Cultural tiket: Discover' your city!
- Unipartys: Let's Celebrate!

Picture

(not printed)

University sports

Sport is fun and healthy...

… and in XYZ you will find great conditios for a sporty lifestyle.

Our sports program has been expanded to include a number of interesting sports in which you, of course, as a student of the University of XYZ, can participate for free.
Look forward to:

Picture (not printed)

+ Full body excercise
+ Power Yoga
+ Women's soccer
+ Ju-Jutsu
+ Hot Iron Workout
+ Strength-endurance-

Picture (not printed)

Further information and all other sports offers can be found on our website:
www.sport-uni-xyz..de

Picture (not printed)

Picture (not printed)

Picture (not printed)

Picture (not printed)

Workschops

Picture (not printed)

Picture (not printed)

Expand your Soft-Skills!

Picture (not printed)

Nowadays Employers expect not only theoretical knowledge and good grades but a convincing appearance is also crucial for the career start. The University of XYZ emphasizes this with a range of workshops, that you can visit at any time during your studies. For example, the following topica are waiting for you:

+ **Presentation techniques**
+ **Moderation and negotiation techniques**
+ **Communication & Interviewing**

We invite you to our welcome event for this topic!

Picture (not printed)

Semester aboad

Picture (not printed)

Picture (not printed)

around the world – Study abroad!

Ever thought of a stay abroad? Take advantage of the many benefits of studying abroad;

Learn the language the country and meet new people, collect experiences for your life and your CV!

The university not only supports studying abroad, but also continuously expands its network of partner universities around the world. We regularly provide informational events on the subject, such as **Erasmus Meet & Greet**. You will be informed of upcoming appointments at the latest in the

first week informed.

We look forward to seeing you?

Picture (not printed)

Here you can write down things that you think can be optimized and that you cannot change:

**Page 1**	**Page 2**	**Page 3**
**Page 4**	**Page 5**	**Page 6**
